# Evaluating community knowledge of tuberculosis preventive therapy in rural South Africa

**DOI:** 10.1186/s12889-025-21719-2

**Published:** 2025-03-07

**Authors:** Carlo Foppiano Palacios, Anthony P. Moll, Roman Shrestha, Tejaswi Kompala, J. Lucian Davis, Salome Charalambous, Lucy Chimoyi, Violet Chihota, Laurie Andrews, Sheela V. Shenoi

**Affiliations:** 1 University of Maryland School of Medicine, Baltimore, USA; 2 Church of Scotland Hospital, Tugela Ferry, South Africa; 3 Philanjalo NGO, Tugela Ferry, South Africa; 4 University of Connecticut, Storrs, USA; 5 University of California School of Medicine, San Francisco, USA; 6 Yale School of Medicine, New Haven, USA; 7 Aurum Institute, Pretoria, South Africa; 8 Cooper Medical School at Rowan University, Camden, USA; 9 School of Public Health, University of Witwatersrand, Johannesburg, South Africa; 10 Division of Infectious Diseases, Vanderbilt University Medical Center, Nashville, USA

**Keywords:** Isoniazid preventative therapy, TB prevention, Rural South Africa

## Abstract

**Background:**

Tuberculosis preventive therapy (TPT) effectively reduces TB incidence among people living with HIV, but implementation remains suboptimal and data on community knowledge of TPT is needed. We sought to understand community members’ knowledge of TB and TPT to facilitate implementation of TPT.

**Methods:**

In rural Msinga, KwaZulu Natal, South Africa, a cross-sectional study was conducted at community events during an HIV and TB testing initiative. Participants ≥ 18 years old who were residents of Msinga were anonymously surveyed. We evaluated Knowledge of TB and TPT, generating separate scores for each domain. Descriptive statistics, chi-square testing, Kruskal-Wallis, linear regression, and exploratory factor analysis (EFA) were performed.

**Results:**

Among 104 respondents, median age was 32.5 years, 65% were female, and 23% completed secondary school. EFA identified two factors for TB knowledge: cultural beliefs of TB’s origin and transmission and understanding TB as a disease. Overall, TB knowledge was poor (median 10, IQR 8-12.5). Over one-third (*N* = 39, 37.5%) were unaware of TPT. Those who had heard of TPT had good knowledge of TPT, with a median score of 4 (IQR 4–4) out of 4. Factors associated with higher TPT knowledge on multivariate linear regression included being motivated to stay healthy to care for one’s family and knowing that TB can be avoided.

**Conclusions:**

Rural South African community members demonstrated poor TB knowledge. Community members with good knowledge of TB were also aware of TPT. Greater community-level public health education and individual-level counseling efforts are needed to facilitate TPT expansion and implementation.

**Supplementary Information:**

The online version contains supplementary material available at 10.1186/s12889-025-21719-2.

## Introduction

Worldwide, tuberculosis (TB) remains the leading cause of death in HIV-positive individuals and is the leading cause of infectious deaths globally [1]. South Africa has one of the greatest HIV and TB burdens worldwide, with an overall TB incidence of 468/100,000 in 2022 [1]. Additionally, TB remains among the top ten causes of death in South Africa annually [1, 2]. The prevalence of latent TB infection is up to 70% in some regions of South Africa [1, 3]. There is a 5–15% lifetime risk of developing active TB disease among those with latent TB infection (LTBI), while the risk increases 20-fold among people co-infected with HIV [4]. 

The World Health Organization (WHO) has identified TB preventive therapy (TPT) as one of its main strategies to reduce the burden of TB in high-incidence settings [1, 5]. TPT effectively reduces TB incidence and mortality, even among those already taking ART [5–7]. While South Africa was among the first to expand TPT implementation among people living with HIV (PLHIV), more recently, only ~ 50% of those eligible for TPT receive it [8]. WHO guidelines previously focused on TPT for children younger than 5 years old who are in contact with a person with active TB and have now expanded those eligible for TPT to include a broader population, including patients with household contacts with TB and those living or working in high TB incidence settings, and endorsed new short-course regimens [9]. There is a need to understand how knowledge about TB and TPT may facilitate or impair uptake and adherence to TPT.

While little is known about community-level knowledge about TB and TB prevention, past studies have found poor knowledge of TB in South Africa. However, these studies have primarily focused on urban areas or high-risk patient populations, while 40–50% of the population lives in rural areas [10, 11]. More recently, a survey from rural South Africa found that 25% had inadequate knowledge about TB, particularly about its causative agents [12]. Prior studies from South Africa identified completion of high school, a history of previous TB, and learning about TB from healthcare workers or teachers as factors associated with TB knowledge [13]. Greater knowledge of TB has been associated with greater TPT uptake and adherence [14, 15], but data on knowledge of TPT is lacking. We sought to understand community members’ knowledge of TB and TPT in a rural, high TB burden setting to inform future TPT expansion and implementation efforts.

## Methods

The study was conducted in the rural Msinga subdistrict of KwaZulu Natal province, home to 180,000 traditional Zulu people. This region is among the poorest in the country, with high unemployment and inadequate access to water [16]. In KwaZulu Natal, 27% of adults ages 15–64 live with HIV, and TB prevalence is extremely high at 737 per 100,000 people as of 2022 [1, 17, 18]. TPT was endorsed by the South African government in 2011 for PLHIV without symptoms of active TB (cough, fever, night sweats, weight loss), regardless of CD4 count, age, and history of TB treatment. At the time of this study, the regimens available for TPT included isoniazid preventive therapy (IPT) given as a daily oral dose for 12 months for PLHIV and six months for non-PLHIV [19]. 

A cross-sectional study was conducted by anonymously surveying a convenience sample of community members following verbal consent at community events as part of a larger HIV and TB testing initiative [20]. Eligible individuals were older than 18 years old and residents of Msinga. Surveys were conducted independently of screening services, and testing results were not linked to survey results. Trained research staff administered surveys in isiZulu in a private, confidential location and requested community members to answer questions assessing their knowledge of TB and TPT. The questionnaire was developed for this study based on knowledge scales aligned with the Information-Motivation-Behavior (IMB) model (Supplement). According to the IMB model, well-informed and motivated individuals can develop behavioral skills to improve practices, such as enhanced adherence to antiretroviral therapy or incorporation of HIV risk reduction behaviors [21]. After the first component of the questionnaire asking about knowledge of tuberculosis, participants were briefly educated by research staff about the high burden of TB in this rural region, risk of TB in all individuals, higher risk among those with HIV disease, government TPT guidelines including daily isoniazid for those with HIV daily for 12 months to reduce the risk of developing active tuberculosis, and that monthly clinic visits to collect medications were needed. After participants completed an initial survey about their knowledge of tuberculosis, staff provided a brief education session regarding the burden of TB, risk factors for acquiring TB, and the use of TPT to prevent TB. After the education session, the survey continued with assessment of willingness to take TPT.

**Fig. 1:**
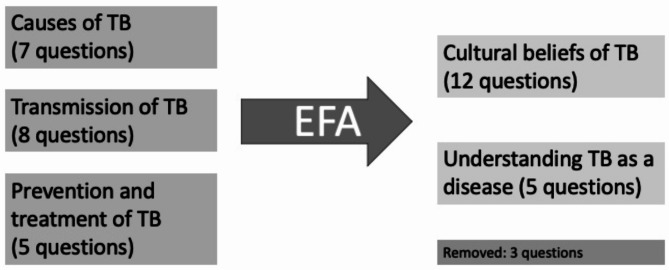
Exploratory factor analysis of original 20 items pertaining to TB and TPT knowledge

Two scores were created corresponding to the number of correct responses to questions regarding the knowledge of TB (20 questions) and TPT (4 questions) using reverse coding (Fig. 1). Each scale was a direct tally of correct responses. Reliability testing was not performed. Operationally, we defined ‘good’ knowledge of TB as 75% correct responses (i.e. 15/20 correct of the TB knowledge items and 3 of 4 for TPT items). We conducted exploratory factor analysis (EFA) to determine which questions to remove and thereby reduce the number of items to calculate each score.

We used descriptive statistics, chi-square, and Kruskal-Wallis testing for the bivariate analysis. For the multivariate analysis, we incorporated all the categories from the bivariate analysis with a p-value ≤ 0.10 into the evaluation of the multivariate linear regression. We performed factor analysis on the TPT knowledge and total TB knowledge scales. We subjected the original four items of the TPT knowledge scale and the original 20 items of the total knowledge of TB score to principal axis factoring and orthogonal varimax rotation with Kaiser. We analyzed all data using SAS version 3.71 and R version 4.0.2.

The suitability of data for factor analysis was assessed for both scales separately before performing the EFA. Regarding the EFA for the total TB knowledge scale, inspection of the correlation matrix revealed the presence of coefficients of 0.3 and above. The overall Kaiser-Meyer-Oklin (KMO) value was 0.718, exceeding the recommended value of 0.5 [22], and Bartlett’s test of sphericity reached statistical significance [23], thus supporting the factorability of the correlation matrix. The initial rotated solution revealed a 6-factor solution that explained 60.13% of the overall variance. Results from the Parallel analysis to determine the optimal number of factors suggested a two-factor solution. Next, we repeated the EFA procedure on the 20 items, forcing the number of factors to be 2. Given three items with loading below 0.40, we eliminated three items, thus leaving 17 items. The same EFA procedure was repeated on the 17 items, resulting in a two-factor solution that explained 38.0% of the variance. Examination of the scree plot suggested that a two-factor solution provided the best fit. The suitability of the two-factor solution was also evident from initial eigenvalues. Factor 1 included 12 items representative of “cultural beliefs of TB” (e.g., “PunishmentGod”; “BeingPoor”; “PunishmentAncestors”; “DrinkingAlcohol”; “MosquitoTB”; “SmokingCigarettes”; “DrinkingTB”; “Witchcraft”; “SexUnprotected”; “ExposureColdAir”; “EatingDrinkingUnclean”; and “ShakingHand.”). Factor 2 contained five items that address “understanding of TB as a disease” (e.g., “TBTreatment6Months”; “PreventiveMedicines”; “SleepingTB”; “BreathingTBAir”; and “AirflowReducesRisk”). The reliability of the two factors was within acceptable to the good range (F1 α = 0.821 and F2 α = 0.636). All the subscales were analyzed as continuous variables, with a higher score indicating a higher degree of knowledge. Next, we performed an EFA for the TPT knowledge scale. Inspection of the correlation matrix revealed the presence of coefficients of 0.3 and above. The Kaiser overall Kaiser-Meyer-Oklin (KMO) value was 0.768, exceeding the recommended value of 0.5 [22], and Bartlett’s test of sphericity reached statistical significance [23], thus supporting the factorability of the correlation matrix. The EFA only revealed a one-factor solution that explained 60.6% of the variance. Examination of the scree plot suggested that a one-factor solution provided the best fit. The suitability of the one-factor solution was also evident from initial eigenvalues. The reliability of the factor was within an acceptable range (F1 α = 0.779). All the subscales were analyzed as continuous variables, with a higher score indicating a higher degree of knowledge.

## Results

### Participant characteristics

A total of 104 community members completed the survey and were included in the analysis. Most participants were women. Median age was 32.5 years (IQR 28–39), and most reported having a partner (Table 1). The majority did not complete secondary school, were unemployed, did not have electricity, and received monthly social welfare grants from the government (~ 350 Rand per month). Most participants traveled for ≥ 30 min to reach the clinic (*N* = 66, 63%), with an average transportation cost of 7.8 Rand (~$0.75 USD). Nearly all participants had previously undergone testing for HIV and TB. Additionally, more than a third (*N* = 37, 36%) reported previous treatment for TB, and 21 participants (20%) reported that someone in their household had previously received treatment for TB.

**Table 1:** Characteristics of respondents (*N* = 104)

Characteristic	*N* (%)
Median age (IQR)	32.5 IQR 28–39
Sex	
Female Male	68 (65.4%) 36 (34.6%)
Marital Status	
Married Reported having a partner Single Widowed	15 (14.4%) 73 (70.2%) 13 (12.5%) 3 (2.9%)
Travel time to clinic	
Less than 30 min 30 min to 1 h 1 to 2 h More than 2 h	65 (62.5%) 35 (33.7%) 3 (2.9%) 1 (1%)
Employed	24 (23.1%)
Received monthly Social Welfare grant	79 (76%)
Completed secondary education	24 (23.1%)
Has electricity at home	35 (33.7%)
Previous Screening	
HIV TB	103 (99%) 87 (83.7%)
TB history	
Previous TB History of household TB	37 (35.6%) 21 (20.2%)
Heard of TPT	65 (62.5%)

### Knowledge of TB

Overall, respondents had poor knowledge of TB (median 10, IQR 8-12.5, max 17), including cultural beliefs of TB (median 5.5, IQR 3–8) and understanding of TB as a disease (median 5, IQR 5–5) as categorized by the EFA analysis (Table 2). For example, 87% thought eating or drinking unclean food or water was a mechanism for acquiring TB.

**Table 2:** Knowledge of TB and TPT in a rural South African community (*N* = 104)

	*N* (%)
Knowledge of TB	
Cultural beliefs about TB	
Witchcraft Drinking alcohol Smoking cigarettes Being poor Punishment by God Punishment by ancestors Mosquito bite Eating or drinking unclean water or food Unprotected sex Exposure to cold air Drinking from the same cup Shaking hands	7 (6.7) 34 (32.7) 68 (65.4) 21 (20.2) 12 (11.5) 5 (4.8) 57 (54.8) 90 (86.5) 56 (53.8) 49 (47.1) 62 (59.6) 30 (28.8)
Understanding TB as a disease	
Breathing air from a person with TB Sleeping in the same room TB treatment takes 6 months Airflow reduces risk of TB transmission Would take preventative medicines	102 (98.1) 93 (89.4) 98 (94.2) 94 (90.4) 94 (90.4)
Knowledge of TPT	
TPT reduces risk of TB TPT needs to be taken for 6 months HIV has a higher risk of TB disease Taking TPT prevents TB	92 (88.5) 92 (88.5) 96 (92.3) 96 (92.3)

### Knowledge of TPT

Over one-third (39, 37.5%) of community members were unaware of TPT. Knowledge of TPT after the educational session was assessed with a median score of 4 (IQR 4–4, max 4). On bivariate analysis (Table 3), knowledge of TPT was significantly associated with multiple factors, including knowing that TB can be avoided, wanting to take care of one’s family, being interested in taking medications to prevent TB, and being willing to take TPT even if protection from TB would only last two years.

**Table 3:** Correlates of TB preventive therapy knowledge

Variable	Unadjusted *p*-value	Unadjusted aOR (95%CI)	Adjusted *p*-value	Adjusted aOR (95%CI)
Agreeing to take medicines to stay healthy even if asymptomatic	0.02	4.41 (2.0-9.7)	-	-
Avoiding missing pills	0.009	3.31 (1.94–5.63)	-	-
Aware of TPT	0.002	1.70 (1.2–2.43)	-	-
Interested in taking medications to avoid TB	0.02	38.61 (7.04-211.76)	-	-
Knowing HIV increases the risk of TB	0.01	2.5 (1.19–5.27)	-	-
Knowing medicine can prevent TB	0.004	8.61 (2.49–29.76)	-	-
Knowing TB can be avoided	0.01	15.6 (4.06-60.0)	< 0.001	16.18 (4.6–57.2)
Knowing TB treatment lasts minimum 6 months	0.003	8.88 (2.83–27.84)	-	-
Not wanting to take any new medications	0.02	1.82 (1.01–3.29)	-	-
Wanting to take care of their family	0.001	14.63 (4.5-47.53)	0.006	5.95 (1.7-21.04)
Willing to take TPT even if they are only protected from TB for 2 years	0.03	3.74 (1.32–10.58)	-	-

### Regression analysis

Multivariate linear regression analysis of knowledge of TPT identified two factors that were associated with higher knowledge of TPT, including being motivated to stay healthy to take care of one’s family (*p* = 0.006, aOR 5.95, 95% CI 1.68–21.04) and knowing that TB can be avoided (*p* < 0.001, aOR 16.18, 95% CI 4.58–57.18) (Table 3).

On linear regression, we found an association between higher total knowledge of TB scores and higher TPT knowledge scores (*p* = 0.04, aOR 2.07 95% CI 1.04–4.11). There was no association between knowledge and travel time to the clinic.

## Discussion

To inform TPT implementation efforts in rural Sub-Saharan Africa, we assessed community-level knowledge of TB and TPT. To our knowledge, our study is the first to evaluate correlates of TPT knowledge among general community members in a high TB prevalence region. We found that community members in our impoverished rural setting had poor knowledge about TB diagnosis and transmission, that about a third of community members were entirely unaware of TPT, and that motivation to stay healthy and care for one’s family was associated with greater knowledge of TPT. To improve future TB knowledge, communities should be better educated about TB, with a particular emphasis on dispelling myths and misperceptions surrounding TB.

In a high-burden TB setting, community members had poor knowledge of TB. This work considered perspectives of the general population of this rural region and not necessarily a traditionally defined high-risk patient population such as only those living with HIV. However, given the high incidence of TB [24], high proportion of symptomatic individuals who do not seek care [25], and emphasis on implementing TB preventive therapy [26], better knowledge of TB among general community members is critical to TB policy and practice. A recent prevalence study of TB in South Africa found that two-thirds of participants with symptoms concerning for TB did not seek care, indicating a need for expanding public health education of TB, community outreach offering testing, and linkage to care [27]. Our findings are similar to those of a prior study of educated patients from an urban site in India that identified low levels of knowledge about TB transmission [28]. Comparatively, our study was performed in a rural South African community where only 24% of participants completed secondary school.

Next, the EFA demonstrated a distinction between knowledge related to cultural beliefs of TB’s origin and transmission and the understanding of TB as a disease. The first factor, “cultural beliefs of TB,” represents misperceptions regarding the causes and spread of TB, such as ascribing TB to witchcraft, being poor, or mosquitos. The second factor, “understanding of TB as a disease”, demonstrates accurate beliefs about the transmission, treatment, and prevention of TB. This factor likely represents the prevalence of misinformation surrounding the true causes and mechanisms for the transmission of TB.

Furthermore, knowledge of TB is relevant to TPT, as past studies have identified that knowledge of TB causes and transmission correlated with intention to seek care for TPT and with TPT adherence [29, 30]. A substantial proportion of community members were unaware of this preventive strategy; implementation of current South African guidelines will be limited without a broader awareness of preventative therapy. Despite one-third of the study population not being aware of TPT before, they answered with good TPT knowledge after brief education, showing the feasibility of such community-based efforts. Nevertheless, these results emphasize that most community members were unaware of TPT, highlighting the need for greater public health education. With more attention to TB and TPT in the setting of rolling out new TPT shorter course regimens, knowledge of TB and TPT should be reevaluated. Additionally, we found that correlates of TPT knowledge included knowledge that TB can be avoided and motivation to stay healthy and care for one’s family. Previous studies have shown that caregivers who are motivated by family care needs are more likely to promote good adherence to TPT among their children [29]. 

Prior work has demonstrated that greater knowledge of TPT is associated with completing a full course of TPT [14, 31–33]. Aside from the need for greater public health education about TB prevention, particularly in rural areas, our data also demonstrate that formal education was associated with improved overall TB knowledge. We found that participants had poor knowledge of the causes and transmission of TB. It is unclear whether individuals’ formal education connotes overall health literacy or is specific to TB [34–38]. Regardless, these data emphasize the critical role of education and awareness in improving implementation [39, 40]. In addition to strengthening public and individual awareness of TPT, future efforts should include ascertaining the impact of education and knowledge levels on TPT retention and treatment completion.

We recognize several limitations. First, while the study was done in the context of community-based HIV testing efforts, we did not have access to individual HIV testing data for this study, and thus, we could not stratify knowledge by HIV status [20]. Despite this, given that more than one-third had previously been treated for TB and one-fifth had household contacts with TB, this study’s results indicate that education and counseling about TB in the general population needs to be strengthened. The most recent WHO guidelines expand the pool of those eligible to receive TPT to general community members who may be in contact with people with TB, making this study particularly relevant for informing widespread implementation. Next, we focused on IPT and did not incorporate items about short-course regimens such as 3HP. Furthermore, we did not assess knowledge of HIV in this sample or the relationship between HIV and TPT. Additionally, this study took place in a rural South African community and may not be generalizable to urban settings. However, the results contribute to the understanding that poor knowledge of both TB and TPT plague resource-limited settings in sub-Saharan Africa. Next, we acknowledge a smaller sample size as reflected in the wider confidence intervals; future studies may consider larger sample sizes to identify subtle differences in TB and TPT knowledge. Lastly, even though a validated knowledge instrument was not used, we performed EFA to support our knowledge questionnaire; future research should seek to develop and apply validated instruments.

## Conclusion

Community members in rural South Africa had poor overall knowledge of TB, particularly about the causes and transmission of TB, and a low level of awareness about TPT. Knowledge of TPT was associated with the motivation to stay healthy and care for one’s family. Greater community-level public health education and individual-level pre-treatment counseling efforts will be needed to facilitate TPT expansion and implementation efforts.

## Electronic supplementary material

Below is the link to the electronic supplementary material.

Supplementary Material 1

## Data Availability

The datasets used and analyzed during the current study are available from the corresponding author on reasonable request.

## Abbreviations

EFA: Exploratory factor analysis
IMB: Information-Motivation-Behavior
IPT: Isoniazid preventive therapy
KMO: Overall Kaiser-Meyer-Oklin
PLHIV: People living with HIV
TPT: TB preventive therapy
TB: Tuberculosis
WHO: World Health Organization
