# Supplementary material for: Evaluating community knowledge of tuberculosis preventive therapy in rural South Africa
Source: BMC Public Health. 2025 Mar 7;25:918. doi: 10.1186/s12889-025-21719-2 (PMC11889810; doi:10.1186/s12889-025-21719-2)
Supplement: Supplementary file 1 — Supplementary Material 1 [file 12889_2025_21719_MOESM1_ESM.docx]

Supplement

Knowledge of TB Questionnaire:

- What causes TB?
- Witchcraft
- Drinking alcohol
- Smoking cigarettes
- Being poor
- Punishment from God
- Punishment from the ancestors
- How do you get TB?
- Exposure to cold air
- Eating or drinking unclean food or water
- Breathing air that a person with TB has coughed into
- Drinking from the same cup as someone with TB
- Sleeping in the same room as someone with TB
- Shaking the hand of someone with TB
- Having sex without a condom
- From a mosquito that has bitten someone with TB
- Do you believe…
- TB treatment takes at least 6 months?
- Good airflow with open windows and fans can reduce risk of TB transmission?
- I would take medicines to stay healthy even if I feel fine?

Knowledge TPT Questionnaire:

- Do you agree with the following statements?
- TPT reduces the risk of getting sick from TB disease.
- TPT is a medicine that needs to be taken every day for six months.
- If you have HIV, there is a higher risk of getting sick with TB.
- If I take TPT, I will not get sick with TB for some time.
